# Salvia miltiorrhiza improves Alzheimer's disease: A protocol for systematic review and meta-analysis

**DOI:** 10.1097/MD.0000000000021924

**Published:** 2020-09-04

**Authors:** Ying Guo, Xing Dong, Renyan Zhang, Yanmei Zhong, Peng Yang, SanYing Zhang

**Affiliations:** a School of Basic Medical Sciences; b School of Medical Information Engineering, Chengdu University of Traditional Chinese Medicine; c Innovative Institute of Chinese Medicine and Pharmacy of Chengdu University of Traditional Chinese Medicine; d Rehabitation Department of Chengdu Fifth People's Hospital, Chengdu, Sichuan, China.

**Keywords:** Alzheimer's disease, protocol, randomized controlled trials, Salvia miltiorrhiza meta-analysis, systematic review

## Abstract

**Background:**

Alzheimer's disease (AD) is an age-related neurodegenerative disease that is slowly becoming a global problem. Salvia miltiorrhiza (SM) has a history of thousands of years of use in China. In recent years, SM has been reported to have the effect of improving Alzheimer's disease. However, there is no systematic review of its efficacy and safety yet. Therefore, we propose a systematic review to evaluate the efficacy and safety of SM for AD patients.

**Methods:**

Six databases will be searched: China National Knowledge Infrastructure (CNKI), China Biological Medicine (CBM), China Scientific Journals Database (CSJD), Wanfang database, PubMed, and EMBASE. The information is searched from January 2010 to July 2020. Languages are limited to English and Chinese. The primary outcomes include changes in the Mini-Mental State Examination (MMSE), Alzheimer's Disease Assessment Scale-cognitive subscale (ADAS-Cog) and Activities of Daily Living scale (ADL). Additional outcomes include clinical effective rate and adverse event rate. The Grading of Recommendations Assessment, Development and Evaluation (GRADE) system will be used to assess the strength of the evidence.

**Results:**

This systematic review will evaluate the efficacy and safety of SM in the treatment of Alzheimer's disease.

**Conclusion:**

This systematic review provides evidence as to whether SM is effective and safe for Alzheimer's disease patients.

**Systematic review registration:**

INPLASY202070066.

## 1 Introduction

Alzheimer's disease (AD) is an age-related neurodegenerative disease characterized by progressive cognitive and memory disorders. It is estimated that there are nearly 47 million AD cases globally, with substantial new cases each year.^[1]^ Attempts to develop therapies for AD patients have not been successful to date.^[2]^ The pathogenesis of AD is very complicated, and the current mainstream view believes that it involves cholinergic system, cell autophagy, neuroinflammation, β-amyloid precursor protein and oxidative stress.^[3–5]^

Salvia miltiorrhiza (SM) is a medicinal plant with a history of more than 2000 years of use in China. Traditional Chinese medicine (TCM) uses SM to treat palpitation, stroke, perimenopausal syndrome, anemia, and other diseases.^[6–8]^ Many clinical trials have found that SM has a significant improvement effect on Alzheimer's disease.^[9–11]^

Unfortunately, there is currently no systematic review of the safety and effectiveness of SM in the treatment of AD. Therefore, we propose a protocol for a systematic review to evaluate the effectiveness and safety of SM in the treatment of AD patients, so as to provide a rigorous evaluation of the existing evidence.

## 2 Methods

### 2.1 Study registration

This protocol of systematic and meta-analysis review has been registered on Inplasy (https://inplasy.com/) with number INPLASY202070066. Ethical approval is unnecessary because this study only involves the data of published previous studies.

### 2.2 Eligibility criteria

#### 2.2.1 Type of study

Only randomized controlled trials (RCTs) can be included. Observation studies, animal research, case report, review, and meta-analysis are excluded.

#### 2.2.2 Participants

Patients diagnosed with AD (using any recognized diagnostic criteria), such as Diagnostic and Statistical Manual of Mental Disorders (DSM-IV), or Recommendations from the National Institute on Aging-Alzheimer's Association work groups on diagnostic guidelines for AD (NIA-AA), or Chinese Guidelines for the Diagnosis and Treatment of Alzheimer's Disease or Other Dementia. There is no restriction on age, gender, nationality of the patient, and the duration and severity of the disease.

Include:

1. vascular dementia, frontotemporal, or any other forms of dementia,
2. other disorders such as Parkinson's disease, traumatic brain injury, stroke, and cancer that may impact cognitive function will be excluded.

#### 2.2.3 Interventions

Analyzed interventions included Salvia miltiorrhiza used as monotherapy, Chinese herbal compound prescription and related alternative therapies.

#### 2.2.4 Comparison

In the same study, AD patients received other treatments.

#### 2.2.5 Outcome measures

The primary outcomes include changes in the Mini-Mental State Examination (MMSE), Alzheimer's Disease Assessment Scale-cognitive subscale (ADAS-Cog), and Activities of Daily Living (ADL) scale. Additional outcomes are clinical effective rate and adverse event rate.

### 2.3 Information source

We search the following databases from January 2010 to July 1, 2020: China National Knowledge Infrastructure, China Biological Medicine, China Scientific Journals Database, Wanfang database, PubMed, and EMBASE. We will search English and Chinese articles for review, and collect additional references from review references and original research articles.

### 2.4 Search strategy

Two review authors will search the literature independently with cross-check. Any inconsistency will be solved by a third reviewer. Manual search will be performed if relevant literatures are found in the included studies. The electronic search will be conducted using a combination of following keywords: AD, Alzheimer's disease, dementia, senile dementia, cognitive impairment, neurocognitive disorder, cognitive, decline, salvia miltiorrhiza, salvia, dan shen, randomized controlled trial, controlled clinical trial, trial, RCT, randomized, randomly. The search strategy for PubMed is presented in Table 1 and the strategy will be modified upon the requirement of other databases.

**Table 1:**
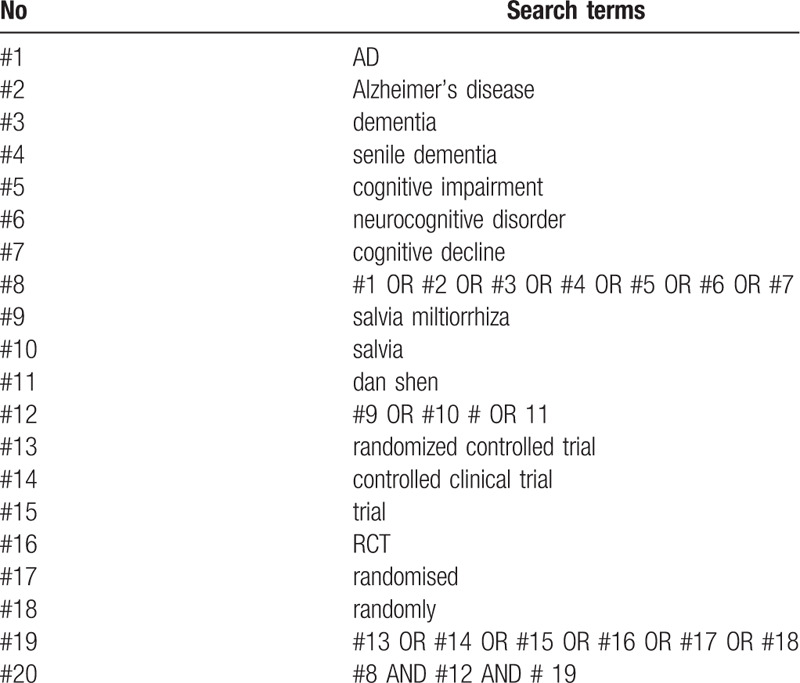
Search strategy for the PubMed.

### 2.5 Data collection and analysis

#### 2.5.1 Study selection

**Figure 1:**
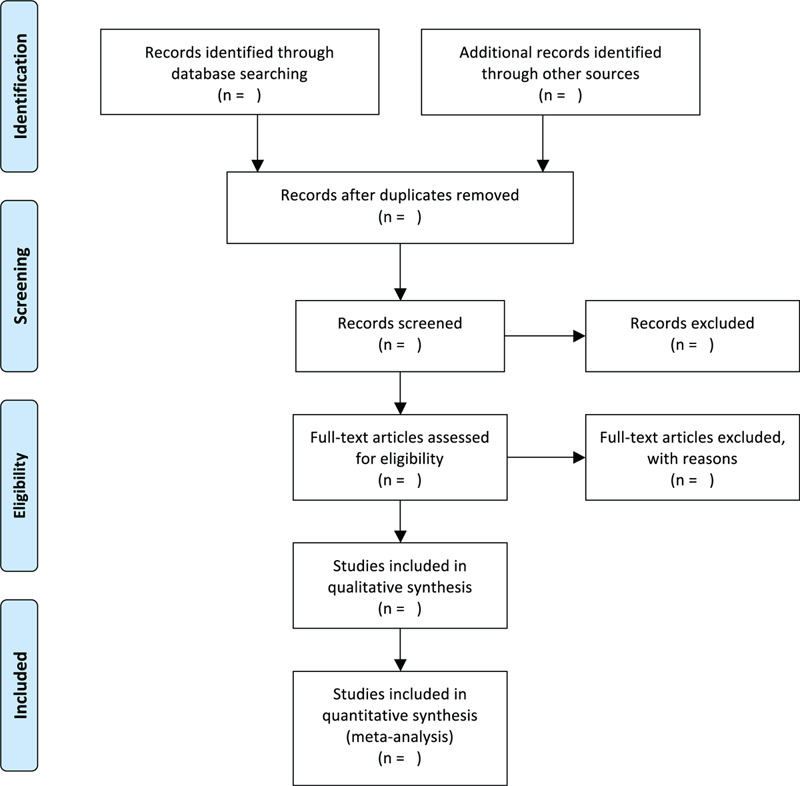
Flowchart of study selection.

Two reviewers will perform literature screening, study selection, and data extraction independently. The literature obtained will be imported into EndnoteX9 to screen the title and abstract, the duplication and studies failing to meet the pre-specified inclusion criteria will be excluded. After reading the full text of the remained literature and discussing within the group, the final included studies will be determined. The corresponding author of original RCT will be contacted when the full text is unavailable. Disagreements will be solved by consulting a third-party arbitrator or discussing within a group. The flowchart of studies searching process is shown in Figure 1.

#### 2.5.2 Data extraction and management

For each RCT, we extracted the following information:

1. general information, including name of the first author and year of publication;
2. participant characteristics, including sample size, AD severity, gender composition, mean age, and diagnostic criteria;
3. intervention details, including the experimental medicine and control group care, doses of medications, duration of treatment; and
4. outcome measures and intergroup differences.

Extract data from the primary and secondary results for further evaluation. The inconsistency between the two reviewers will be resolved by the third reviewer.

#### 2.5.3 Risk of bias in included studies

The quality of the studies will be assessed by using the Cochrane Handbook 5.1.0 (Cochrane Handbook 5.1.0). The assessment will include random sequence generation, randomization correctness, allocation scheme hiding, blinding of patients and implementers, accuracy of data results, and other risk of bias. The risk of low bias is expressed as “low risk” and the risk of high bias is expressed as “high risk.” The information provided in the studies is inaccurate or does not provide sufficient information for the bias assessment to be expressed as “unclear risk.” The above content evaluation was independently evaluated by 2 researchers, and any differences will be resolved through discussions with the third reviewer.

#### 2.5.4 Measurement of treatment effect

Two reviewers will analyze the data independently using RevMan 5.3.5 Risk ratio with 95% confidence interval will be adopted for the dichotomous data, whereas the mean difference or standardized mean difference with 95% CI will be utilized for the continuous data.

#### 2.5.5 Assessment of reporting biases

A funnel plot will be performed to assess any publication bias when more than 10 RCTs are included. In additional, Egger regression and Begg correlation test will also be performed to identify the funnel plot asymmetry.

#### 2.5.6 Assessment of heterogeneity

The Cochrane *I*^2^ and *Q* tests will be applied to evaluate the heterogeneity with the cut-off value of *I*^2^ = 50. If *I*^2^ > 50% and/or *Q* test < 0.10, the heterogeneity will be deemed significant.

#### 2.5.7 Data synthesis

In line with the Cochrane guideline, a fixed-effect model will be utilized to pool and analyze the outcome data if *I*^2^ < 50, and a random-effect model will be employed if *I*^2^ ≥ 50. Subgroup analysis or meta-regression will be performed to assess the potential sources and present reasonable explanations for the heterogeneity.

#### 2.5.8 Sensitivity analysis

Sensitivity analysis will be applied to evaluate the stability of the pooled results of included RCTs according to the methodological quality, sample size, and missing data.

#### 2.5.9 Grading the quality of evidence

The Grading of Recommendations Assessment, Development and Evaluation (GRADE) guidelines will be utilized to grade the quality of evidence as very low, low, moderate, or high.

## 3 Discussion

AD is an age-related neurodegenerative disease characterized by progressive cognitive and memory disorders, senile plaques, neurofibrillary tangles, and neuronal cell death.^[12]^ According to the Alzheimer's disease report, there are more than 40 million AD patients in the world, and as the problem of aging increases, the number of AD patients will continue to increase.^[13]^ AD as the main cause of dementia accounts for the total 70% of patients.^[14]^ However, effective cure method for AD and dementia is still absent, and mainstream research currently focuses on reducing progressive clinical symptoms rather than its cure.^[15]^ TCM is a unique treatment, which has been in use since 2000 years ago in China. It differs from modern medicine and has its own distinctive advantages.^[16,17]^ The use of natural herb is a characteristic of TCM. Salvia miltiorrhiza first recorded in the *Shennong's Classic of Materia Medica,* is widely distributed in East Asia. TCM uses SM to treat palpitations, stroke, perimenopausal syndrome, anemia, and other diseases. Previous research found that Salvia miltiorrhiza has anti-inflammatory, anti-oxidation, and anti-fibrosis effects.^[18–20]^ A growing body of studies have shown that Salvia miltiorrhiza can alleviate cognitive dysfunction of AD patients, indicating that it may be a useful therapeutic agent for AD.^[21–23]^ The way SM improves AD is to reduce the accumulation of β-amyloid protein, reduce the oxidative stress response, and improve the energy system of the cholinergic system.

However, currently no systematic review and meta-analysis have been conducted regarding the efficacy and safety of SM for the treatment of patient with AD. The findings of this study may yield helpful evidence for the clinicians and investigators concerned in decision-making process about the efficacy and safety of SM for patients with AD.

## Acknowledgments

The authors would like to thank SanYin Zhang for critically reviewing the manuscript.

## Author contributions

**Data collection:** Ying Guo, Xing Dong.

**Statistical analysis:** Renyan Zhang, Yanmei Zhong.

**Supervision:** SanYin Zhang, Peng Yang.

**Writing – original draft:** Ying Guo.

**Writing – review & editing:** SanYin Zhang.

## Correction

When originally published, Dr. Sanyin Zhang's name appeared incorrectly as San Ying Zhang. This has since been corrected.

## References

[1] Anstey KJ Peters R Clare L Joining forces to prevent dementia: the International Research Network on Dementia Prevention (IRNDP). Int Psychogeriatr 2017;29:1757 60. 28899450 10.1017/S1041610217001685 PMC5873600

[2] Babulal GM Quiroz YT Albensi BC Perspectives on ethnic and racial disparities in Alzheimer's disease and related dementias: update and areas of immediate need. Alzheimer's Dement 2019;15:292 312. 30555031 10.1016/j.jalz.2018.09.009 PMC6368893

[3] Sugarman MA Alosco ML Tripodis Y Neuropsychiatric symptoms and the diagnostic stability of mild cognitive impairment. Journal of Alzheimer's disease: JAD 2018;62:1841 55. 29614641 10.3233/JAD-170527 PMC6548196

[4] Van Cauwenberghe C Van Broeckhoven C Sleegers K The genetic landscape of Alzheimer disease: clinical implications and perspectives. Genet Med 2016;18:421 30. 26312828 10.1038/gim.2015.117 PMC4857183

[5] Rajmohan R Hemachandra Reddy P Amyloid-beta and phosphorylated tau accumulations cause abnormalities at synapses of Alzheimer's disease neurons. J Alzheimer's Dis 2017;57:975 99. 27567878 10.3233/JAD-160612 PMC5793225

[6] Li Z-M Xu S-W Liu P-Q Salvia miltiorrhizaBurge (Danshen): a golden herbal medicine in cardiovascular therapeutics. Acta Pharmacol Sin 2018;39:802 24. 29698387 10.1038/aps.2017.193 PMC5943903

[7] Zhou L Zuo Z Chow MSS Danshen: an overview of its chemistry, pharmacology, pharmacokinetics, and clinical use. J Clin Pharmacol 2005;45:1345 59. 16291709 10.1177/0091270005282630

[8] Hung YC Pan TL Hu W-L Roles of reactive oxygen species in anticancer therapy with Salvia miltiorrhiza Bunge. Oxidat Med Cell Long 2016;2016:5293284. 10.1155/2016/5293284 PMC4989081 27579153

[9] Ren B Liu Y Zhang Y Tanshinones inhibit hIAPP aggregation, disaggregate preformed hIAPP fibrils, and protect cultured cells. J Mater Chem B 2018;6:56 67. 32254193 10.1039/c7tb02538f

[10] Wang Q Yu X Patal K Tanshinones inhibit amyloid aggregation by amyloid-( peptide, disaggregate amyloid fibrils, and protect cultured cells. ACS Chem Neurosci 2013;4:1004 15. 23506133 10.1021/cn400051e PMC3756451

[11] Jiang P Li C Xiang Z Tanshinone IIA reduces the risk of Alzheimer's disease by inhibiting iNOS, MMP-2 and NF-(Bp65 transcription and translation in the temporal lobes of rat models of Alzheimer's disease. Mol Med Rep 2014;10:689 94. 24859152 10.3892/mmr.2014.2254

[12] Coyle JT Price DL DeLong MR Alzheimer's disease: a disorder of cortical cholinergic innervations. Science 1983;219:1184 90. 6338589 10.1126/science.6338589

[13] Alzheimer's Association Report. 2020 Alzheimer's disease facts and figures. Alzheimers Dement, 2020, undefined: undefined.

[14] Alzheimer's Association. 2016 Alzheimer's disease facts and figures. Alzheimers Dement, 2016, 12: 459–509. 10.1016/j.jalz.2016.03.001 27570871

[15] Shah RC Bennett DA Physicians and Alzheimer dementia: past, present, and future. Ann Intern Med 2020;172:695 6. 32340036 10.7326/M20-1500

[16] Ren L Wang J Feng L Efficacy of Suxiao Jiuxin Pill on coronary heart disease: a meta-analysis of randomized controlled trials. Evid Based Complement Altern Med 2018;2018:9745804. 10.1155/2018/9745804 PMC5892298 29770157

[17] Wang J Wong YK Liao F What has traditional Chinese medicine delivered for modern medicine? Expert Rev Mol Med 2018;20:e4. 29747718 10.1017/erm.2018.3

[18] Jiang P Guo Y Dang R Salvianolic acid B protects against lipopolysaccharide-induced behavioral deficits and neuroinflammatory response: involvement of autophagy and NLRP3 inflammasome. Journal of Neuroinflammation 2017;14:239. 29212498 10.1186/s12974-017-1013-4 PMC5719935

[19] Chen F Li L Tian D-D Salvia miltiorrhiza roots against cardiovascular disease: consideration of herb-drug interactions. Biomed Res Int 2017;2017:9868694. 28473993 10.1155/2017/9868694 PMC5394393

[20] Forouzanfer F Hosseinzadeh H Medicinal herbs in the treatment of neuropathic pain: a review. Iran J Basic Med Sci 2018;21:347 58. 29796216 10.22038/IJBMS.2018.24026.6021 PMC5960749

[21] Miroddi M Navarra M Quattropani MC Systematic review of clinical trials assessing pharmacological properties of Salvia species on memory, cognitive impairment and Alzheimer's disease. CNS Neurosci Ther 2014;20:485 95. 24836739 10.1111/cns.12270 PMC6493168

[22] Cai H Lian L Wang Y Salvia miltiorrhiza Protective effects of injection against learning and memory impairments in streptozotocin-induced diabetic rats. Experimental and Therapeutic Medicine 2014;8:1127 30. 25187809 10.3892/etm.2014.1919 PMC4151631

[23] Ma X Xu W Zhang Z Salvianolic acid B ameliorates cognitive deficits through IGF-1/Akt pathway in rats with vascular dementia. Cell Physiol Biochem 2017;43:1381 91. 28992623 10.1159/000481849

